# 

**Published:** 1899-07

**Authors:** 


### PERSONALS.

Representative C. E. Wells, veterinary surgeon, of Macomb, was an active spirit in the Michigan State Legislature the past winter, and was zealous in all measures of interest to horsemen generally.

Dr. H. H. Weinberg and John H. McNeal, graduates of the Veterinary Department of the University of Pennsylvania, were matriculates at the Medical Department of the Medico-Chirurgical College, in Philadelphia, for the years '98 and '99.

Dr. H. L. Ramacciotti has been appointed Veterinarian to the Greater American Exposition of 1899, to be held in Omaha, and will gladly welcome any of the profession during this period. Dr. Ramacciotti expects to be present in New York in September.

Dr. John Robertson, late veterinary surgeon of the 2d Cavalry of the United States Army, and recently second Lieutenant of the 6th Infantry, has been promoted to first lieutenant of the 6th Infantry, and was among the wounded while on duty in Cuba.

Dr. F. C. Grenside, of New York City, has located at New port, R. I., for the summer season.

Dr. John S. Meyer, formerly of St. Joseph, Mo., is now a resident of Challis, Idaho, but no longer a devotee of the profession.

Dr. Leonard Pearson was a visitor to New York and Boston the latter part of June.

Dr. Victor A. Norgaard, of Washington, D. C , was a visitor to New York City in June, spending a portion of the time as the guest of Dean Gill, of the New York College of Veterinary Surgeons.

Dr. M. E. Conard, of West Grove, Pa., spent a period in June at Atlantic City, N. J., listening to what the wild waves were saying.

Dr. Corcoran, senior veterinarian of the Eighth Cavalry, has returned from Cuba, and is in a hospital in New York City.

Dr. Frank A. Avrill, formerly of Fullerton, is now located at Wisner, Neb.

Dr. J. N. Megary, of Baltimore, Md., a graduate of the Veterinary Department of the University of Pennsylvania, has been pursuing a course of medical studies at the University of Maryland; he was a visitor to Philadelphia in June to renew his allegiance among his fellow alumni.

Dr. Leonard Pearson, of Philadelphia, was a visitor to Boston in the early part of January.

Dr. John W. Adams, of the Veterinary Department of the University of Pennsylvania, addressed a Farmers' Institute at West Grove, Pa.

Dr. W. L. Rhoads, of Lansdowne, Pa., corresponding secretary of the Pennsylvania State Veterinary Medical Association, addressed a Farmers' Institute at Manoa, in January.

Dr. Leonard Pearson was a visitor to Washington in May, and spent the evening with the District of Columbia Veterinary Association.

Dr. H. M. Hunter, formerly of Tulare, Cal., has removed to Visalia, same State.

The senior class of the Veterinary Department of the University of Pennsylvania made visits to the Walker-Gordon dairies and several prominent dairy farms in Chester County during the month of April, to familiarize themselves with the systems used in producing milk for the market under the most advanced sanitary measures.

